# Association of Duration of Sleep and Cardiovascular and Metabolic Comorbidities in Sleep Apnea Syndrome

**DOI:** 10.1155/2012/316232

**Published:** 2011-12-29

**Authors:** Zeynep Zeren Ucar, Ali Kadri Cirak, Serhan Olcay, Hatice Uysal, Ahmet Ugur Demir, Rıfat Özacar

**Affiliations:** ^1^The Department of Sleep Disorders, Dr Suat Seren Chest Diseases and Surgery Training and Research Hospital, Yenisehir, 35110 Izmir, Turkey; ^2^The Department of Pulmonary Diseases, Dr Suat Seren Chest Diseases and Surgery Training and Research Hospital, 35110 Izmir, Turkey; ^3^The Department of Pulmonary Diseases, Faculty of Medicine, Hacettepe University, 06100 Ankara, Turkey

## Abstract

*Background/Aim*. Previous population-based studies found association between duration of sleep and cardiovascular and metabolic comorbidities. Our aim was to investigate the association between the duration of sleep and cardiovascular and metabolic comorbidities in OSAS. *Patients and Methods*. The study enrolled 312 patients, who had polysomnography (PSG) during 2006-2007 and responded to a telephone-administered questionnaire providing information on characteristics of sleep on average 12 months after PSG. *Results*. Of the patients, 90 were female (28.8%), 173 (58.5) received the diagnosis of OSAS, 150 (45%) had no comorbidities, 122 had hypertension (HT), 44 had diabetes mellitus (DM), and 38 had coronary heart disease (CHD). Mean ± SD of age in years was 47.2 ± 10.6, 56.5 ± 9.3, 53.2 ± 8.9, and 59.9 ± 9.0 for the no comorbidity, HT, DM, and CHD groups, respectively. Reported duration of sleep was not associated with any of the comorbidities in the overall group. In the analysis restricted to OSAS patients, sleep duration ≤6 hours was significantly associated with CHD after the adjustment for age, gender, and other associated factors (OR: 5.8, 95% CI: 1.0–32.6). *Conclusions*. Confirmation of the association between shorter duration of sleep and CHD will provide prognostic information and help for the management of OSAS.

## 1. Introduction

Sleep loss is a common condition in modern society. Although the health effects of sleep deprivation have been obscure, recent epidemiological studies have revealed relationships between sleep deprivation and hypertension (HT), coronary heart disease (CHD), and diabetes mellitus (DM) [1]. Because sleep deprivation increases sympathetic nervous system activity, this increased activity serves as a common pathophysiology for HT, DM, and CHD. Previous studies showed that sleep duration less than 6 hours or more than 8 hours is associated with increased morbidity and mortality due to cardiovascular diseases in the general population [2, 3].

Obstructive sleep apnea syndrome (OSAS) is a common medical disorder that is growing in prevalence worldwide. It is characterized by recurrent cycles of intermittent hypoxia and there is increasing evidence that intermittent hypoxia plays a role in the development of cardiovascular risk in OSAS patients through the activation of inflammatory pathways. Some excellent review articles have already summarized the effects of OSAS on HT, CHD, and DM [4–6]. The pathogenesis of cardiovascular disease in OSAS is not completely understood but is likely to be multifactorial, involving a diverse range of mechanisms including sympathetic nervous system overactivity, endothelial dysfunction, and selective activation of inflammatory molecular pathways [7–9]. Expanding our understanding of these pathways, which include chronic intermittent hypoxia and provocation of inflammation by sleep deprivation, will yield novel therapeutic targets with the scope of reducing the cardiovascular risk in OSAS.

Hence, both sleep deprivation and OSAS are related to the occurrence and consequences of cardiovascular and metabolic diseases. To our knowledge, there have been no reports on the relationship between sleep duration and risk of comorbidities in OSAS patients. Our aim was to investigate the association between duration of sleep and cardiovascular and metabolic comorbidities in patients admitted to sleep lab with sleep apnea symptoms and in patients who received the diagnosis of OSAS.

## 2. Material and Methods

### 2.1. Patients and Study Protocol

**Figure 1:**
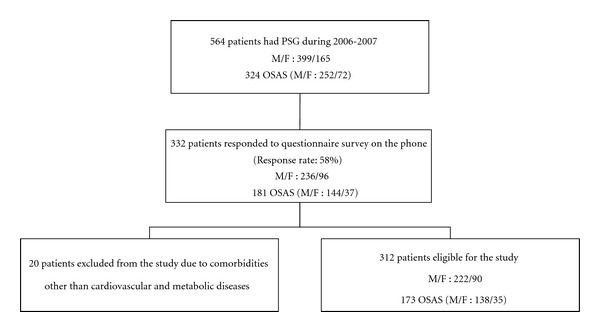
Flowchart of the study, describing the study population: PSG: polysomnography, M: male, F: female, OSAS: obstructive sleep apnea syndrome defined as excessive daytime sleepiness (Epworth Sleepiness Scale score >10) and AHI (apnea-hypopnea index) ≥5 or AHI ≥ 15, regardless of excessive daytime sleepiness.

Of the 564 patients, who had polysomnography during 2006-2007 with a preliminary diagnosis of sleep apnea in the sleep center, 332 (response rate: 58%) gave consent and responded to a telephone administered survey on average 12 months after polysomnography (PSG), which included information on characteristics of sleep and physician diagnosis of systemic diseases. The study protocol was approved by the Institutional Review Board of the research hospital. Cardiovascular and metabolic diseases (comorbidities) included hypertension, diabetes mellitus, and coronary heart disease. Patients were grouped in hypertension, diabetes mellitus, and coronary heart disease, and no comorbidity groups according to their responses. In the condition of reporting more than one of these diseases, the patient was grouped in all these disease groups; that is, the groups were not exclusive. Twenty patients, who reported none of these three diseases (DM, HT, and CHD), but a systemic disease including neuromuscular diseases, heart failure, and chronic obstructive lung diseases were omitted from the analysis. Thus, the study population comprised of 312 subjects (55% of the original sample).  Figure 1 describes flowchart of the study population. There was no significant difference between the age, gender, and OSAS percentage of the patients in the study population and the patients who did not respond to the telephone administered survey.

Duration of sleep was assessed by the question: “On average how many hours do you sleep at night?” In the analysis, duration of sleep was categorized as 6 hours or less, 7-8 hours, and more than 8 hours, based on the previous studies, which suggested 7 hours of sleep as normal.

### 2.2. Polysomnography

PSG was performed in the Sleep Laboratory with Grass Technologies Comet Series EEG/PSG with AS40 Amplifier System running Grass Technologies Twin software version 4 (Grass Technologies, Astro-Med Inc. Product Group, USA) and included four electroencephalography (EEG) channels (C3 to A1, C4 to A2, O1 to A2, and O2 to A1), right and left electrooculography (EOG) channels, one chin electromyography (EMG) channel and four tibialis anterior EMG channels, finger pulse oximeter, strain gauges for thoracoabdominal movements, one electrocardiography (ECG) lead, a nasal airflow (pressure cannula), a nasal thermistor and a digital microphone for snoring detection. PSG recordings were scored in 30-second epochs for sleep, breathing and oxygenation according to the standard criteria of the American Academy of Sleep Medicine (AASM) [10]. Obstructive apnea was defined as a 90% cessation of oro-nasal airflow for at least 10 seconds in the presence of chest-wall motion. Hypopnea was defined as a reduction in the airflow of 50% or more associated with 3% or more arterial oxygen desaturation and/or arousal, or a reduction in respiratory airflow of 30% or more associated with 4% or more arterial oxygen desaturation and/or arousal for at least 10 seconds. Apnea hypopnea index (AHI) was calculated as the total number of apneas and hypopneas per hour of sleep. Diagnosis of OSAS was based on PSG findings, according to the International Classification of Sleep Disorders 2 (ICSD-2) [11], as in the following:

- Excessive daytime sleepiness and AHI ≥ 5;
- AHI ≥ 15, regardless of excessive daytime sleepiness.

Excessive daytime sleepiness was defined as the score of Epworth sleepiness scale >10 [12]. Subjects were classified in the control group if they had AHI < 5 regardless of excessive daytime sleepiness.

### 2.3. Statistical Analysis

Descriptive statistics and comparison of the comorbidity groups with the no comorbidity group are shown in the tables. Frequency (percentage) and mean ± standard deviation were used for the descriptive statistics of the categorical and continuous variables, respectively. Chi-square testing was used for the univariate analysis of categorical variables. Fisher's exact test was used in the case of an expected frequency of less than five in 25% of the cells. Student's *t*-testing and Mann-Whitney *U* test were used for the univariate analysis of continuous variables, where appropriate (normal and not-normal distribution, resp.). Logistic regression analysis models were constructed to adjust the association between duration of sleep and comorbidities for age, gender, BMI, other potential risk factors, and factors which were found significant in the univariate analysis. Models were constructed both in the overall group and in the OSAS group, to investigate the association between duration of sleep and comorbidities both in the overall study population and in OSAS patients. *P* value less than 0.05 was used to define statistical significance in the two-sided tests.

## 3. Results

The study enrolled 312 patients, who had PSG with a preliminary diagnosis of sleep apnea between 2006 and 2007 and answered a telephone administered questionnaire providing information on characteristics of sleep.  Table 1 shows the personal characteristics of the subjects in the cardiovascular and metabolic comorbidity groups. Of the subjects 90 were female (28.8%), 150 (45%) had no comorbidity, 122 had HT (39.1%), 44 had DM (14.1%), and 38 had CHD (12.1%). Diagnosis of OSAS was made in 173 (58.5%) of the patients, without a significant difference between the groups of HT (60.2%), DM (48.7%), and CHD (48.4%) and no comorbidity group (58.5%). Of the OSAS patients 138 were men and 35 were women. Coronary heart disease group compared to no comorbidity group had a higher percentage of less educated patients. In the PSG record, time percentage spent <90% of oxygen (T90%) was significantly higher in the HT group than no comorbidity group.  Table 2 shows the association between PSG findings and cardiovascular and metabolic comorbidity groups.

Of the patients 58 reported onset of sleep delayed (delayed sleep onset) more than 30 minutes (18.5%), 211 had fragmented sleep (67.6%), 174 had unrefreshening sleep (55.7%), and 78 had excessive daytime sleepiness defined as ESS score above 10 (25%). There was no significant difference in the prevalence of sleep symptoms between comorbidity groups and no comorbidity group, except for the significantly higher percentage of delayed sleep onset in the HT group than the no comorbidity group (25.4% versus 13.3).

**Figure 2:**
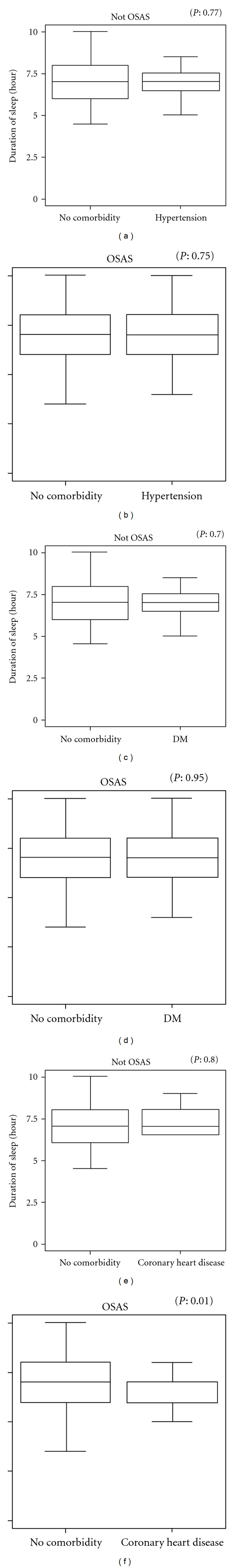
Boxplot graph of the distribution of duration of sleep in HT, DM, and CHD comorbidity groups compared to no comorbidity group in the subjects without OSAS (Not-OSAS) in graphs a, c and e and in the subjects with OSAS (OSAS) in graphs b, d and f: HT: hypertension, DM: diabetes mellitus, CHD: coronary heart disease, and OSAS: obstructive sleep apnea syndrome. Mann-Whitney *U* test was used in the comparisons.

Duration of sleep was reported as 6 hours or less in 15%, >6 hours to 7 hours in 25.2%, >7 hours to 8 hours in 47.9%, and >8 hours in 11.8% of the subjects. There was no significant difference between the sleep duration of the comorbidity groups and the no comorbidity group.  Figure 2 shows the boxplot graph of the distribution of duration of sleep in HT, DM, and CHD groups compared to no comorbidity group in patients with and without OSAS. Among the OSAS patients, duration of sleep was significantly shorter in the CHD group than in the no comorbidity group.

Table 3 shows the association between reported sleep duration and cardiovascular and metabolic comorbidity groups in the models adjusting for other risk factors. In the whole group duration of sleep was not associated with any of the comorbidities. In the analysis restricted to OSAS patients, sleep duration of ≤6 hours was significantly associated with coronary heart disease as unadjusted and after the adjustment for age, gender, smoking status, and educational status.

## 4. Discussion

In this study, we have demonstrated that shorter duration of sleep (6 hours or less) was associated with CHD in patients with OSAS after adjustment for confounding factors in patients with OSAS.

To our knowledge, this is the first study which examined the association between sleep duration and cardiovascular and metabolic comorbidities in patients with OSAS. Previous studies found significant association between short (less than 6 hours) and long sleep duration (more than 8 hours) and CHD, DM, and HT in general population [2, 3].

Recent studies including follow-up data have shown that OSAS has a causal relationship with the development of cardiovascular disease independent of confounding factors such as sex, age, and obesity [13–22]. The mechanisms by which OSAS exerts its detrimental effects remain to be established, and future studies should actively pursue the identification of OSAS patients at high risk of cardiovascular diseases. Epidemiological studies have convincingly shown that type 2 diabetes is often associated with OSAS and daytime sleepiness [23–25]. For example, in a large series of OSAS patients, type 2 diabetes and impaired glucose tolerance showed a 30 and 20% prevalence, respectively [26], and studies in snorers reached similar conclusions [27, 28]. There is strong evidence that OSAS is an independent risk factor for systemic hypertension [29, 30]. Case-control studies have confirmed the association between sleep apnea and increased blood pressure independent of confounders such as obesity [31]. Severity of OSAS (AHI, ESS, and T90%) was not associated with DM and CHD in our study. Percentage of night time spent with oxyhemoglobin saturation below 90% was higher, and difficulty in initiating sleep was more frequent in patients with HT.

The pathogenesis of cardiovascular disease in OSAS is not completely understood but likely to be multifactorial, involving a diverse range of mechanisms including sympathetic nervous system overactivity, selective activation of inflammatory pathways, endothelial dysfunction, hypercoagulability, and metabolic dysregulation, the latter particularly involving insulin resistance and disordered lipid metabolism. Such a clinical context makes it difficult to assess the independent effects of OSAS on cardiovascular risk, and still there remains factors to be understood [32, 33].

The literature supports the view that short or long sleep duration is independently associated with an increased likelihood of coronary events [2, 34–39]. Increased unadjusted CHD risk was found in both short sleeper groups (extremely short ≤5 h, and 6 h sleepers) and extremely long sleepers (≥10 h) in both genders when compared with midrange (7-8 h) sleepers. It was also reported as an important finding that 9-hour sleepers did not differ in their CHD risk when compared with midrange sleepers in the same study [40]. Short sleep duration (<6 h) has been shown as a significant risk factor for coronary events in a Japanese male working population [41]. The risk of CHD events was independent of prominent cardiovascular risk factors and occupational factors [41]. Short sleep duration imposed on a group of healthy subjects increased sympathetic nervous system activity and blood pressure elevation. Therefore, sustained short sleep duration could lead to adverse cardiovascular consequence. We investigated the association between reported sleep duration and cardiovascular and metabolic comorbidities in the models adjusting for the other risk factors including obesity and smoking [42–44]. In these models, only CHD in OSAS patients was associated with shorter sleep duration. Since a few subjects reported sleep duration of 10 hours or more, we could not investigate the association between longer sleep time and CHD.

The strengths of the study were examination of both PSG data and self-reported sleep duration and their association with comorbidities. We defined no comorbidity group as patients who did not report the investigated diseases and other systemic diseases. However, these patients might have comorbidities (which were not diagnosed yet), and this misclassification, if nondifferential, could be underestimating the association between sleep duration and comorbidities.

The present study had some limitations. The major limitations of the study were small sample size, which was a constraint for the adjustment of the potential confounders in the analysis. Since this is a cross-sectional study it is difficult to interpret the causality of our findings. However, there is not enough reason to believe that comorbidities strongly affected the duration of sleep. Sleep duration was only associated with CHD in the OSAS group. If comorbidities had strongly changed the sleep duration, this association must have also been observed in DM and HT. We did not have data about the other confounders like hyperlipidemia, alcohol intake, and exercise frequency and therefore could not control these factors in the analysis confounders. Objective measurements of sleep duration, blood pressure, and glucose were lacking, which is another limitation of our study. The prevalence of comorbidities in the control group was not significantly different from the patients with OSAS, which suggests that selection bias (i.e., higher degree of suspicion and screening for systemic diseases and thus finding higher prevalence of systemic diseases in OSAS patients) is not so likely. In this study, reported sleep duration and sleep symptoms were used as an estimate of the general status of sleep habits and sleep symptoms. Patients who were using CPAP therapy were asked to report the situation (duration of sleep and sleep symptoms) before treatment. We did not find a beneficial effect of CPAP compliance on the sleep-related symptoms, which could be due to questions asking the subjects about their symptoms before treatment. Duration of sleep and quality of sleep might have changed due to comorbidities, and this could influence the interpretation of the associations found in this study. Lack of association between sleep duration and other comorbidities (HT and DM) could be regarded as evidence against this possibility. Importantly, the control group consisted of patients who were admitted to the sleep center with sleep-related complaints, including snoring and poor quality of sleep. Previous studies which used snoring as a proxy for sleep-related breathing disorders have found significant association between snoring and cardiovascular diseases [45, 46]. Thus, the control group in this study was not representing the healthy individuals in the general population. This could, at least partially explain the lack of association between comorbidities and duration of sleep and OSAS, in the study population. Presence of sleep symptoms (excessive daytime sleepiness, unrefreshening sleep, or difficulty in initiating sleep) was not associated with the comorbidities in the regression analysis. This could be due to the high prevalence of these symptoms in the no comorbidity group (which was almost similar to that of comorbidity groups). The only significant association was found between CHD and duration of sleep in the analysis restricted to OSAS patients. This could suggest the interaction between OSAS and short duration of sleep for the development of CHD. Confirmation of this finding requires follow-up data.

In conclusion, CHD was associated with shorter sleep duration in patients with OSAS in the present study. Our results, if confirmed, suggest that ensuring appropriate sleep hours through appropriate OSAS treatment and modifying lifestyle factors and sleep hygiene could be beneficial in the prevention of cardiovascular and coronary events in patients with OSAS.

## Figures and Tables

**Table 1:** Demographic features of the patients.

	*N*	No comorbidity	HT	DM	CHD
*N*	*312*	150	122	44	38
Male/Female, (%)	*222/90*	116/34 (77.3)/(22.7)	76/46** (62.3)/(37.7)	29/15 (63.4)/(36.6)	28/10 (74.3)/(25.7)
Age, (year)		47.2 ± 10.6	56.5 ± 9.3***	53.2 ± 8.9**	59.9 ± 9.0***
BMI, (kg/m^2^)		30.0 ± 4.8	32.5 ± 6.0***	32.3 ± 5.9*	30.6 ± 4.8
Educational status					
Primary or less, %	*149*	43.9	51.3	47.5	82.4***
Secondary, %	*98*	36.6	31.9	27.5	**11.8**
University, %	*59*	19.6	16.8	25.0	**5.9**
Smoking status					
Nonsmoker, %	*120*	36.7	46.3	36.6	31.4
Ex-smoker, %	*94*	34.7	24.0	36.6	20.0
Current smoker, %	*97*	28.7	29.8	26.8	48.6
Shift work, %	*73*	23.3	21.7	31.7	25.7

Means and ±SDs are given in the table, unless otherwise specified.

*P < 0.05;

**P < 0.01;

***P < 0.001.

Significant findings are marked in bold type.

HT: hypertension, DM: diabetes Mellitus, CHD: coronary heart disease, and OSAS: obstructive sleep apnea syndrome.

**Table 2:** Polysomnography findings and features of OSAS of the patients in different cardiovascular and metabolic comorbidity groups.

	*N*	No comorbidity	HT	DM	CHD
*N*	*312*	150	122	44	38
Total sleep time, min		385.4 ± 82.1	346.5 ± 88.6**	335.8 ± 87.0**	348.5 ± 98.9
T90%, (%)		13.4 ± 21.8	22.8 ± 30.8*	23.6 ± 5.0	16.2 ± 26.8
Sleep efficiency, (%)		87.5 ± 10.5	84.5 ± 10.5*	84.0 ± 13.3	84.8 ± 11.4
WASO, (%)		2.2 ± 6.3	4.3 ± 8.4*	4.0 ± 9.1	3.5 ± 7.6
REM, (%)		11.3 ± 6.4	9.5 ± 7.1*	10.4 ± 5.8	11.3 ± 7.4
N3, (%)		21.8 ± 12.4	21.0 ± 14.6	16.4 ± 5.8*	20.0 ± 15.0
AHI, (/hour)		30.3 ± 27.9	33.1 ± 30.7	31.3 ± 31.5	22.4 ± 22.2
Obstructive AHI		27.6 ± 26.4	31.9 ± 30.1	27.1 ± 27.9	19.9 ± 19.0
OSAS, %	*173*	58.5	60.2	48.7	48.4
Severe OSAS, %	*120*	38.5	38.0	34.1	27.8
CPAP prescribed, %	*116*	33.3	37.7	48.8	38.9
CPAP compliance, %	*61*	52.0	55.6	60.0	42.9

Means and ±SDs are given in the table, unless otherwise specified.

*P < 0.05;

**P < 0.01.

Significant findings are marked in bold type.

HT: hypertension, DM: diabetes mellitus, CHD: coronary heart disease, ESS: Epworth Sleepiness Scale, OSAS: obstructive sleep apnea syndrome, and WASO: wake after sleep onset.

OSAS: excessive daytime sleepiness and AHI ≥ 5 or AHI ≥ 15, regardless of excessive daytime sleepiness.

Severe OSAS was defined as AHI above 30/h.

CPAP compliance: reported usage of CPAP every night for more than 4 hours a night. CPAP compliance percentage was calculated in the patients who were prescribed CPAP therapy.

**Table 3:** Association between reported sleep duration and cardiovascular and metabolic comorbidities in the models adjusting for other risk factors.

	Whole group		OSAS patients	
Comorbidities and duration of sleep	Unadjusted OR (95% CI)	Adjusted OR (95% CI)	Unadjusted OR (95% CI)	Adjusted OR (95% CI)
HT				
6 hours or less	1.0 (0.6–1.8)	0.6 (0.3–1.3)	1.3 (0.6–2.7)	0.7 (0.2–1.7)
8.5–10 hours	1.0 (0.4–2.2)	0.7 (0.3–1.8)	1.4 (0.5–4.4)	0.8 (0.2–2.7)
DM				
6 hours or less	1.6 (0.7–3.5)	1.2 (0.5–3.0)	2.7 (0.8–8.5)	2.3 (0.7–7.8)
8.5–10 hours	1.7 (0.6–4.8)	1.2 (0.4–3.7)	3.7 (0.9–15.9)	2.3 (0.4–11.4)
CHD				
6 hours or less	1.1 (0.5–2.9)	1.2 (0.4–3.8)	**3.6 (1.1–11.3)**	**5.8 (1.0–32.6)**
8.5–10 hours	0.4 (0.1–2.1)	0.3 (0.06–2.3)	NA	NA

Adjustments were made for OSAS, age, gender, and BMI in all models, and additionally for smoking status in HT and for smoking status and educational status in CHD.

For duration of sleep, 7-8 hours was considered as the reference category.

Significant findings are marked in bold type.

HT: hypertension, DM: diabetes mellitus, CHD: coronary heart disease, and OSAS: obstructive sleep apnea syndrome.
